# Structural and molecular basis of angiotensin-converting enzyme by computational modeling: Insights into the mechanisms of different inhibitors

**DOI:** 10.1371/journal.pone.0215609

**Published:** 2019-04-18

**Authors:** Li Fang, Mingxian Geng, Chunlei Liu, Ji Wang, Weihong Min, Jingsheng Liu

**Affiliations:** 1 College of Food Science and Engineering, Jilin Agricultural University, Changchun, China; 2 National Engineering Laboratory of Wheat and Corn Deep Processing, Changchun, China; 3 Changchun Vocational Institute of Technology, Changchun, China; Universidade Nova de Lisboa Instituto de Tecnologia Quimica e Biologica, PORTUGAL

## Abstract

Angiotensin-I converting enzyme (ACE) is a two-domain dipeptidylcarboxypeptidase involved in regulating blood pressure via the kallikrein-kininand renin-angiotensin-aldosterone complex. Therefore, ACE is a key drug target for the treatment of cardiovascular system diseases. At present many works are focus on searching for new inhibitory peptides of ACE to control the blood pressure. In order to exploit the interactions between ACE and its inhibitors, molecular dynamics simulations were used. The results showed that (a) the secondary structures of the three inhibitor-protein complexes did not change significantly; (b) root-mean-square deviation (RMSD), radius of gyration (Rg), and solvent-accessible surface area (SASA) values of Leu-Ile-Val-Thr (LIVT)-ACE complexes were significantly higher than that of other systems; (c) the backbone movement of LIVT was vigorous in Asp300-Val350, compared with that in Tyr-Leu-Val-Pro-His (YLVPH) and Tyr-Leu-Val-Arg(YLVR), as shown by the center-of-mass distance; and (d) the backbone movement of Asp300-Val350 may contribute to the interaction between ACE and its inhibitors. Our theoretical results will be helpful to further the design of specific inhibitors of ACE.

## Introduction

Angiotensin-I converting enzyme (ACE), also called peptidyldipeptidase A (EC 3.4.15.1), belongs to the type-I membrane-anchored dipeptidylcarboxypeptidase family and is involved in controlling blood pressure by regulating electrolyte homeostasis via the reninangiotensin system [1]. ACE is a zinc metallopeptidase that is has little sequence homology with the other members in peptide family [1]. A comparison of ACE with other proteins using the DALI server [2] showed that ACE has obvious homology with neurolysin [3], which is in the M3 family of oligopeptidases, and is similar to a carboxypeptidase of the M32 family of carboxypeptidases from the hyperthermophilic archaeon *Pyrococcus furiosus* [4]. Similar to ACE, neurolysin and carboxypeptidase are all metallopeptidases contained in the HEXXH active-site motif, which mostly has α-helices with very few β-structures.

The human ACE has two functional domains (N and C), each of which has an active site with a zinc ion binding site [5]. The N and C domains have some differences in their substrate specificities, physiological forms, and inhibitors [6]. On one hand, the N and C domains catalyze the hydrolysis of substrates with similar efficiencies. However, it was reported that inhibition of the N domain of ACE has no impact on the regulation of blood pressure [6, 7]. Targeting the C domain was found to be sufficient for controlling blood pressure, and therefore, all inhibitors target this site.

**Fig 1:**
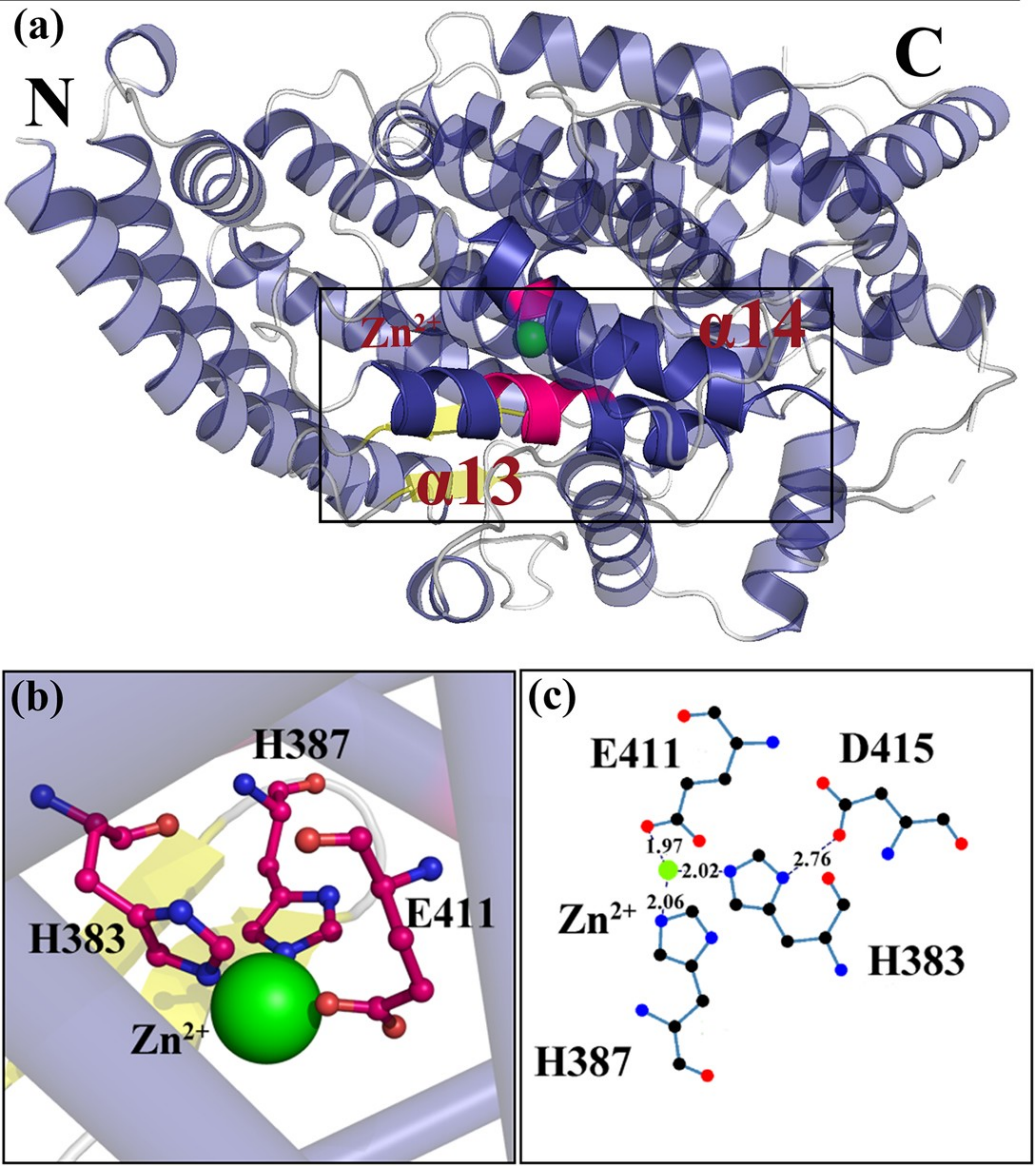
Activity sites of angiotensin-converting enzyme. (a) Organization of the ACE protein. The active sites, located in α13 and α14 of ACE, are indicated by a black rectangle. (b) Zinc-binding motif. The residues that surround the Zn^2+^are represented by pink sticks. (c) The hydrogen bond interaction between Zn^2+^ and key residues. The distance between key residues and zinc is shown by aligplot.

Zinc is an important catalytic component of ACE [1, 8, 9]. As shown in Fig 1, α13 has a zinc-binding motif with two histidine (His383 and His387) coordinated with a zinc ion (+2) [5]. Notably, ACE also can be used as a neurotensin for its wide number of substrates [10].

Through ACE inhibition, the half-life of bradykinin is prolonged and can lead to accumulation and activity [11, 12]. Until now, the exact mechanism of ACE inhibitors in angioedema is yet to be fully explained; but the structural relationship between ACE and inhibitors is clearly implicated.

Previously, we purified and identified two different sources of ACE inhibitory peptides with good activities, YLVPH and YLVR, screened from pine nut and hazelnut, respectively [13, 14]. Based on previous findings, the mechanism of inhibition of ACE by the peptides needs to be further explored. Therefore, another inhibitory peptide, LIVT [15], with high activity was selected from the literature. These three ACE inhibitors (LIVT, YLVPH, and YLVR) (see Table 1) were docked to ACE, and molecular dynamics (MD) simulations were performed to explore the conformational changes for binding of the different inhibitors. Our results provide a theoretical basis for the design of ACE inhibitors as well as insight into the structural and molecular properties of ACE and the mechanisms of inhibition of the different inhibitors.

**Table 1:** Identified bioactive peptides from literature.

Examples of identified bioactive peptide	IC_50_(μM)	Reference
Tyr-Leu-Val-Pro-His	151.0	LiuXQ, MiaoXY, WuD, LiuCL, Fang L, LiuJS and Min WH. (2018)
Tyr-Leu-Val-Arg	15.42	Liu CL, Fang L, Min WH, Liu JS and LiHM. (2018)
Leu-Ile-Val-Thr	0.11	S Vallabha V and Tiku PK. (2014)

## Materials and methods

### Structure preparation

The initial structure of ACE (PDB ID 1O8A) [1] was obtained from the Protein Data Bank. Three protein-ligand complexes in the MD simulations were taken from Autodock4.2. The geometries of YLVPH, YLVR, and LIVT [16] were fully optimized using the B3LYP at the 6-31G* set [17] with the Gaussian 09 software [18].

### Docking study

The energy minimized models of the three inhibitors were used for molecular docking of the substrate onto the target proteins using Autodock tool Version 4.2 [19]. In the 3D structure of ACE, lisinopril, an inhibitor of ACE [1], located at the active pocket. Docking was performed using a pre-set simulation grid box size of 126 × 126 × 126 Å along the X, Y, and Z axes and centered at 39.946, 40.191, and 45.879, in which lisinopril was functioned as the center of the grid box, whereas the targeted docking grid box size was set to 70 × 70 × 60 Å (centered at 43.946, 40.191, 33.879) contained all the active residues (Tyr520, Lys511, His513, His353, Ala354, Glu384, Tyr523, His387, His383, Asp377 and Glu162. The dockings were used for 100 runs using the Lamarckian Genetic Algorithm (LGA). The results were evaluated using root-mean-square deviation (RMSD) values, ligand-protein interactions, and binding energy (ΔGbind) as well as a number of conformations that existed in a populated cluster. The charge values of metal ions have been indicated to play a crucial role in predicting correct docking simulations. The ligand-protein interaction was visualized using Pymol [19] and VMD [20].

### MD simulations

The Amber99SB force field [21] was applied to describe the protein and ligand. The parameterization of the ligand was performed by Antechamber [22]. All complex systems were subjected to MD simulations in a periodic boundary box with the TIP3P water model. Sodium and chloride ions were added to neutralize the simulated systems and to ensure an ion concentration of 150 mM ionic strength. Energy minimization was performed through the steepest descent method. The energy-minimized structure was allowed to reach an initial structure of equilibration. Subsequently, 100 ps NVT and 100 ps NPT were used to maintain the system in a stable environment (300 K, 1 bar). The temperature was increased from 0 to 300 K and maintained at 300 K for 100 ps. All bond lengths were restrained with the LINCS algorithm [23]. After all of the thermodynamic properties had stabilized, the molecular system was simulated for 400 ns with a time step of 2 fs.All coordinates were preserved every 2 ps.

### Computational alanine scanning

Alanine scanning is a tool in protein system that is used to evaluate the contribution of a specific amino acid residue to the stability and function of a protein. This tool is also used to understand whether the side chain of a specific amino acid residue plays a role in the protein’s bioactivity [24]. And so, computational alanine scanning was used with mutations for the active site residues. The binding free energy for the mutated system and the WT system via trajectories of the three protein-ligand complexes (YLVPH, YLVR, and LIVT) for the 10,000 snapshots from the 400 ns trajectory was used for the FoldX approach [25]. The graphical user interface for the FoldX calculations was used [25], which was supplemented as a plugin for the YASARA molecular graphics software [26]. If the free energy change between mutant and wild type denoted as ΔΔG = ΔG (MT)-ΔG (WT) is > 0, the mutation can be seen as destabilizing, while ΔΔG < 0 suggests that the mutation will be stabilized [25–29].

## Results and discussion

### Ligand optimization and docking pose

**Fig 2:**
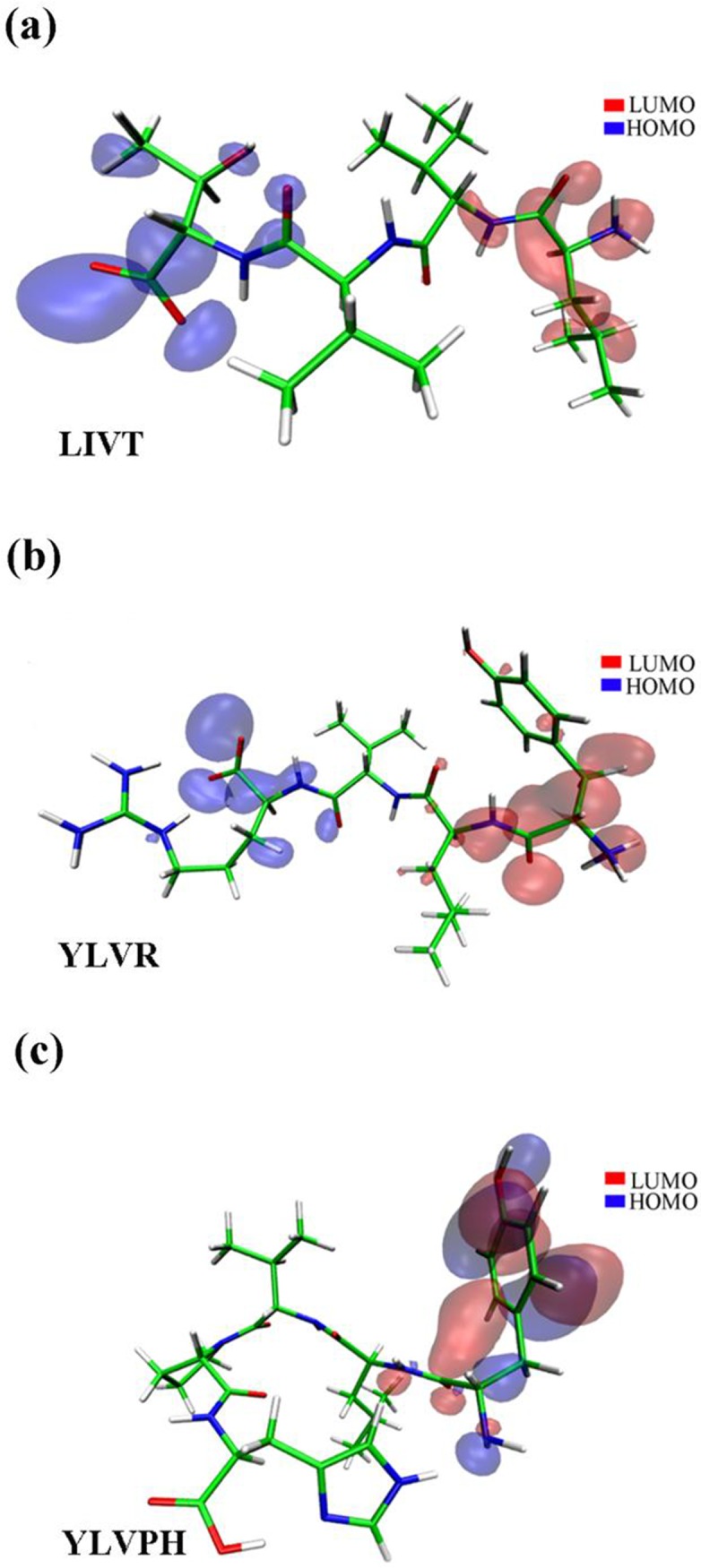
Geometric structure of the inhibitor was optimized by Gaussion09. (a-c) The highest occupied molecular orbital (HOMO) and lowest unoccupied molecular orbital (LUMO) of LIVT, YLVR, and YLVPH are displayed, respectively. The LUMO orbit is colored red and the HOMO orbit is colored blue.

Three reported ACE inhibitors (LIVT, YLRD, and YLVPH) [16] (see Table 1 and Fig 2) were optimized at B3LYP at the 6-31G* level set [17] with Gaussian 09 software [18]. The geometric structures of the three inhibitors may be in the lowest energy state in a natural environment. The highest occupied molecular orbital (HOMO) of the three inhibitors was colored blue, and the lowest unoccupied molecular orbital (LUMO) of the three inhibitors was colored red (Fig 2). The HOMO and LUMO illustrated the chemical reactivity and kinetic stability of the molecules [30], which are essential roles in the study of electrical and chemical properties of molecules and have been used to estimate the bioactivity from intramolecular charge transfer [31].

**Fig 3:**
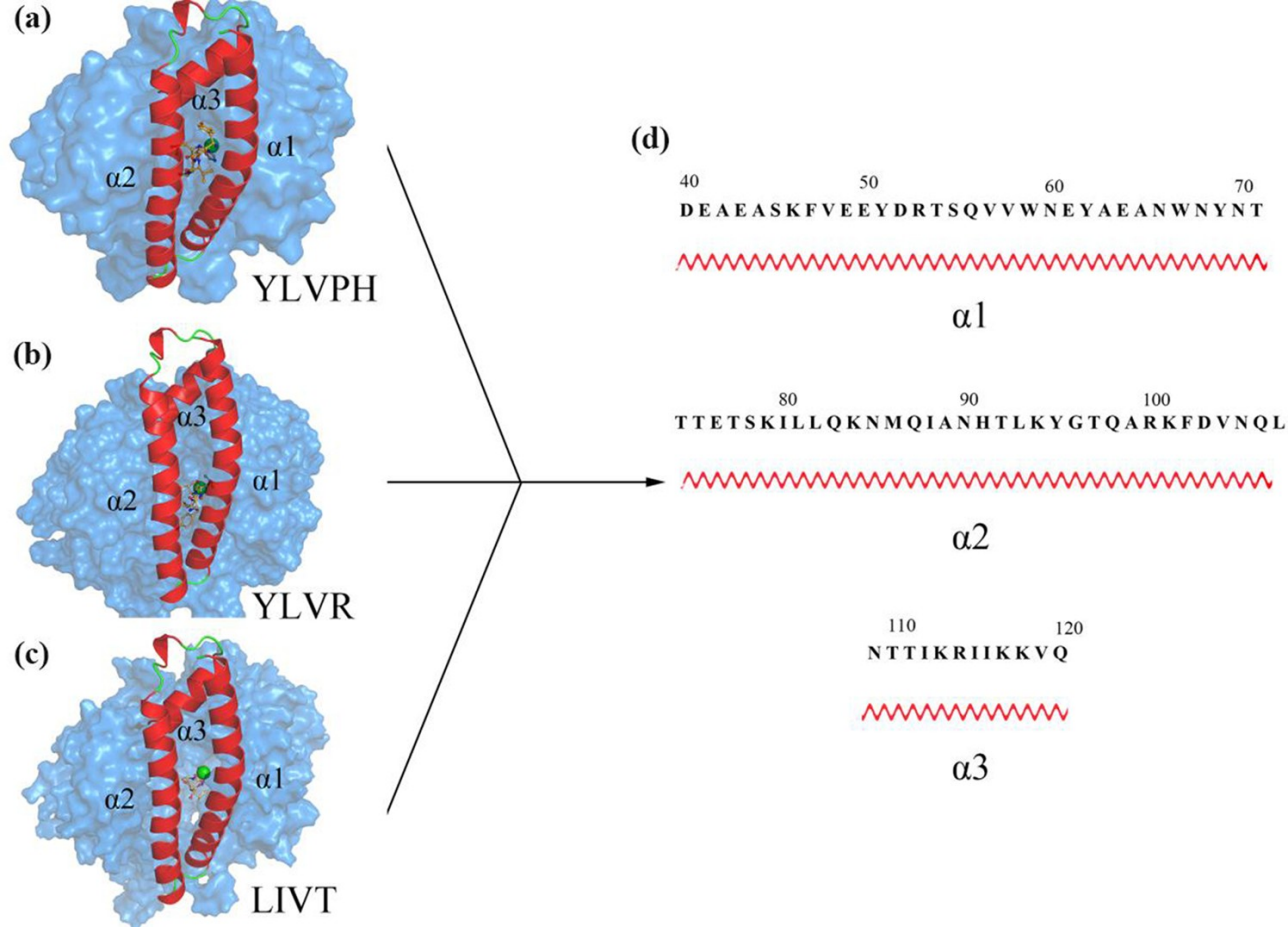
Results of docking between ACE and inhibitors. (a-c) The relative positions of LIVT, YLVR, and YLVPH located at ACE protein, respectively. The α1, α2, and α3 helices, surrounding the inhibitors, are indicated in red. The inhibitor molecules are shown as orange sticks. (d) The primary structure sequence of helix α1, α2, and α3.

**Fig 4:**
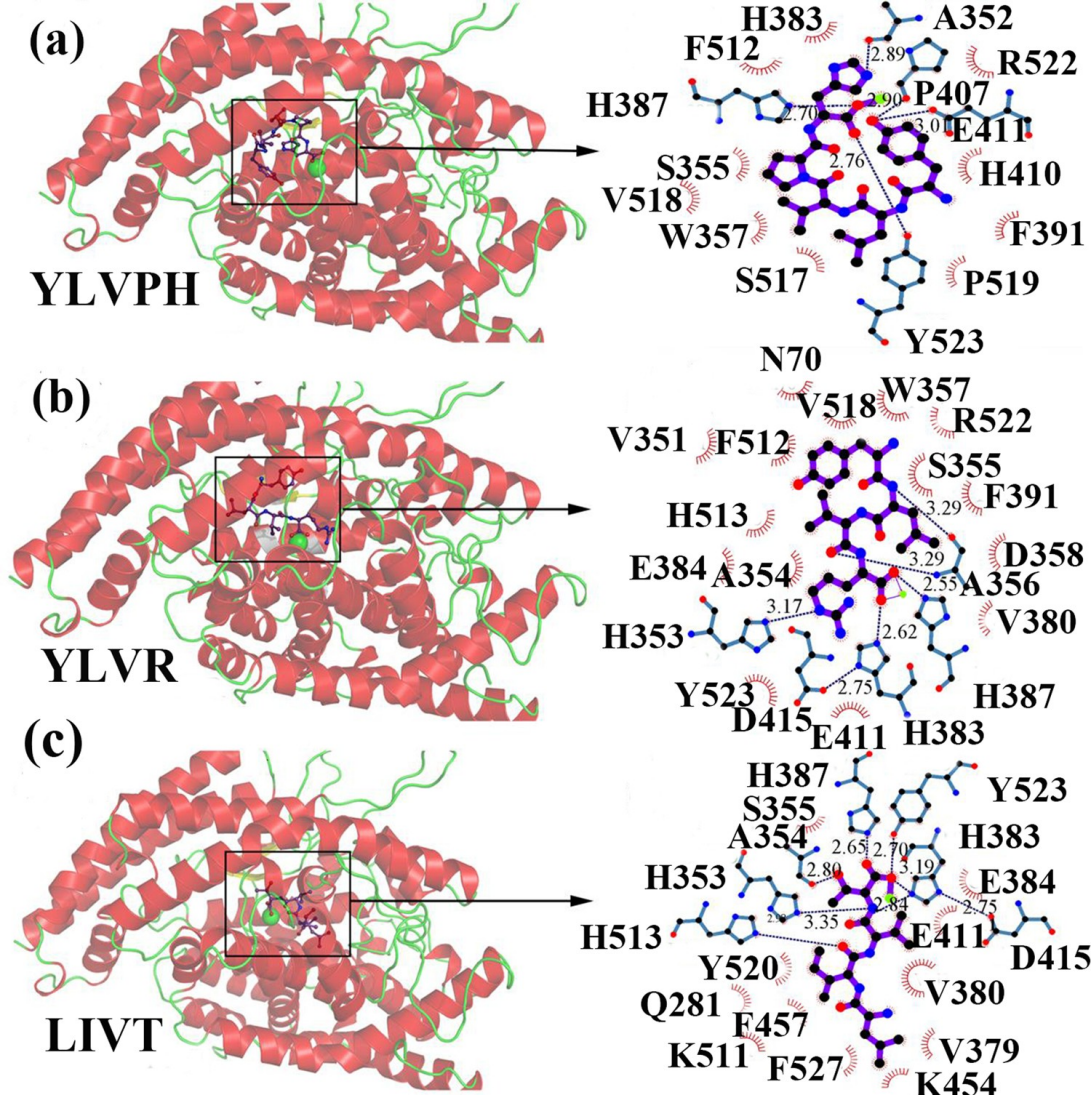
3D plots of the frontier molecular orbital of the three inhibitors. (a-c) Result of binding of the three inhibitors to ACE. The ACE protein is shown as a red cartoon. The hydrogen bonds between protein and inhibitors are indicated by a blue dotted line. The residues that had a weak interaction with inhibitors are colored by the red eyelash.

The three inhibitors were docked to ACE using AutoDock 4.2 [19]. The docking results were listed in S1 Table. The 3D structures of the ACE inhibitors are shown in Fig 3. As shown, the three inhibitors were all located in the active groove, and on top of the molecule there was an N domain ‘lid’ that contained α1 (residues 40–71), α2 (residues 74–107), and α3 (residues 109–120) helices, all of which contained several charged residues (Fig 3). This seems to hinder the access of large polypeptides to the active site, which is the reason for the enzyme’s inability to hydrolyze large-size substrates. 3D plots of the frontier molecular orbital of the three inhibitors are shown in Fig 4.The orbit of ACE was predicted by CHEXVIS [32, 33] (Fig 4), which is the software that can compute the physicochemical properties of the channels with an enzyme family and the mutability of residues in the channels. It can be concluded that the bioactivity of the three inhibitors was different (Ile group of LIVT, Leu group of YLRD, and Tyr group of YLVPH), which may affect the binding affinity of the three inhibitors to ACE.

The docking results from AutoDock 4.2 revealed that YLAPH occupied an active groove surrounded by Ala352 (2.89 Å), Glu411 (3.09 Å and 2.90 Å), Tyr523 (2.70Å), and His387 (2.70Å) (Fig 4). In this binding pose, His387, His512, Ser355, Val518, Trp357, Ser517, Pro519, Pro391, His410, and Arg522 have van der Waals contacts with YLAPH (Fig 4), as calculated by the Ligplot [34]. The binding energy value of YLVPH was -7.45kcal/mol.

The docking results for YLVR with ACE revealed that YLVR also occupied the active groove. However, the binding pose in the active site was different from that of YLVPH. YLVR was lined by His353 (3.17 Å), Ala356 (3.29 Å), His383(2.62 Å), and His387(2.55 Å), and the van der Waals contacts were Asn70, Val518, Trp357, Arg522, Val351, Phe512, and Ser355 (Fig 4). Meanwhile, LIVT occupied the active groove and was surrounded by Ala (2.80 Å), His353 (3.35 Å), His383 (3.19 and 2.84 Å), His387 (2.65 Å), His513 (2.98 Å), and Tyr523 (2.70 Å), and the van der Waals contacts were Ser355, Glu411, Tyr520, Gln281, Phe457, Lys511, Phe527, Lys454, Val379, and Val380 (Fig 4).

The binding energy values of YLVR and LIVT to ACE were -7.03 and -9.94 kcal/mol, respectively. The binding energy indicates stronger interactions of LIVT with ACE, as compared to YLVPH and YLVR. This is the reason why LIVT had a much lower IC_50_ (Table 1) than that of the other inhibitors.

### Structure stability for the three ACE-inhibitor complexes

**Fig 5:**
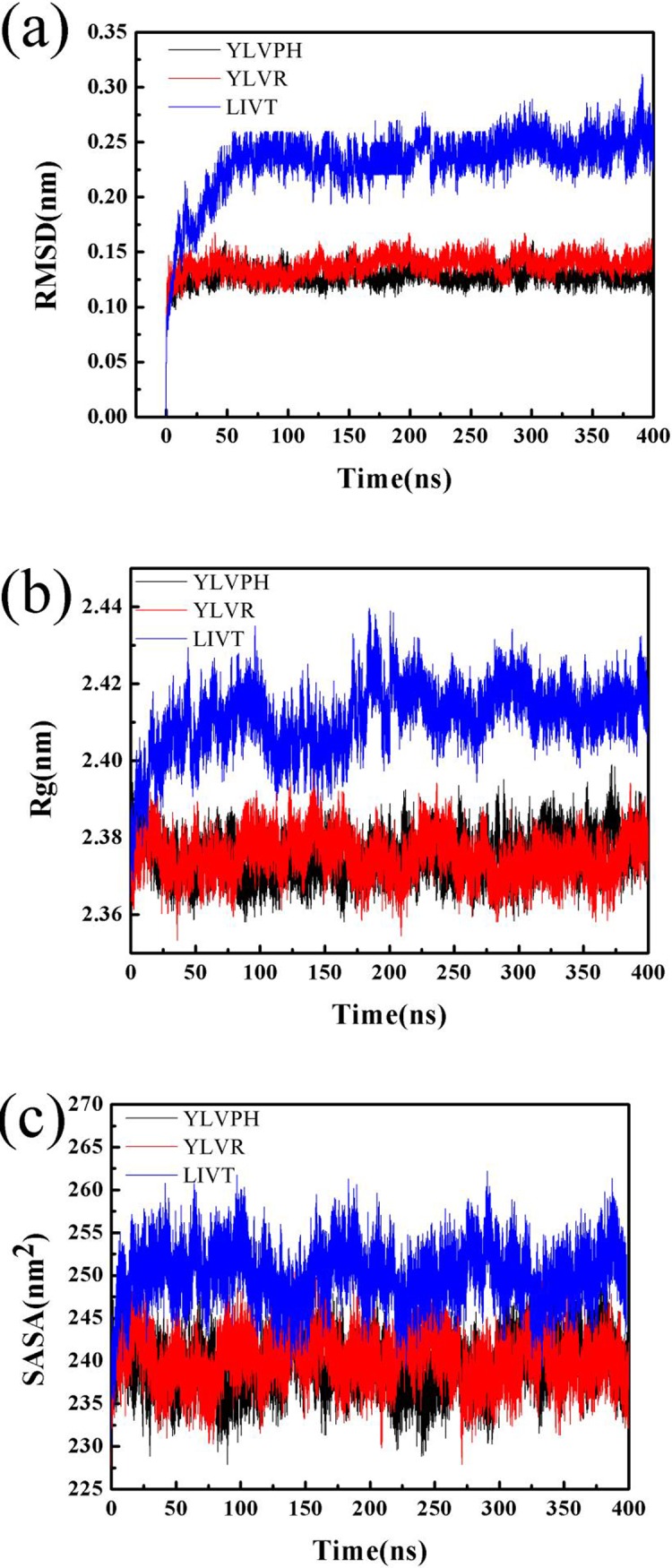
Structural stability analysis of three ACE-inhibitor complexes (ACE-YLVPH, ACE-YLVR, and ACE-LIVT). (a) The plot of root-mean-square deviation (RMSD), (b) the plot of radius of gyration (Rg), (c) and the plot of solvent accessible surface (SASA) for three inhibitor-protein complex systems during 400 ns molecular dynamics (MD) simulations.

Three MD simulations were performed for 400 ns, individually. The position of the inhibitors after the MD simulations remained on the active site (S1 Fig). The structural deviations of Cα atoms during the MD simulation for ACE-inhibitor complexes were calculated each time by an RMSD plot (Fig 5A). The RMSD for ACE-LIVT showed a higher deviation than that of the other two complexes. In contrast, the simulation reached equilibrium after 60 ns for the ACE-LIVT complex, whereas the simulation reached equilibrium after 10 ns for ACE-YLVPH and ACE-YLVR, respectively. The RMSD plot indicated that the complex of ACE-YLVPH and ACE-YLVR rapidly arrived at equilibrium after the initial period, with approximately 0.27 nm deviations. In particular, the RMSD plots for YLVPH and YLVR were about 0.125 nm. The radius of gyration (Rg) refers to several measures related to the size of an object, surface, or ensemble of points. Fig 5B shows the Rg of three ACE-inhibitor complexes. The mean Rg of ACE-LIVT was 2.42 nm, whereas the mean Rg of ACE-YLVPH and ACE-YLVR was about 2.38 nm. The mean Rg for ACE-LIVT was larger than that of the other complexes. This finding may be the reason why LIVT binding to ACE facilitated the conformational rearrangement of the active domain in ACE. This result was in agreement with the experimental data where LIVT had the largest IC_50_ of the three inhibitors (Table 1). Furthermore, the SASA was calculated from the simulations. Fig 5C shows that ACE-LIVT has a larger SASA than that of the two other types. The larger the SASA value, the greater the hydrophobicity. The increased hydrophobicity of residues may facilitate contact with LIVT.

### Conformational changes of the three ACE-inhibitor complexes

**Fig 6:**
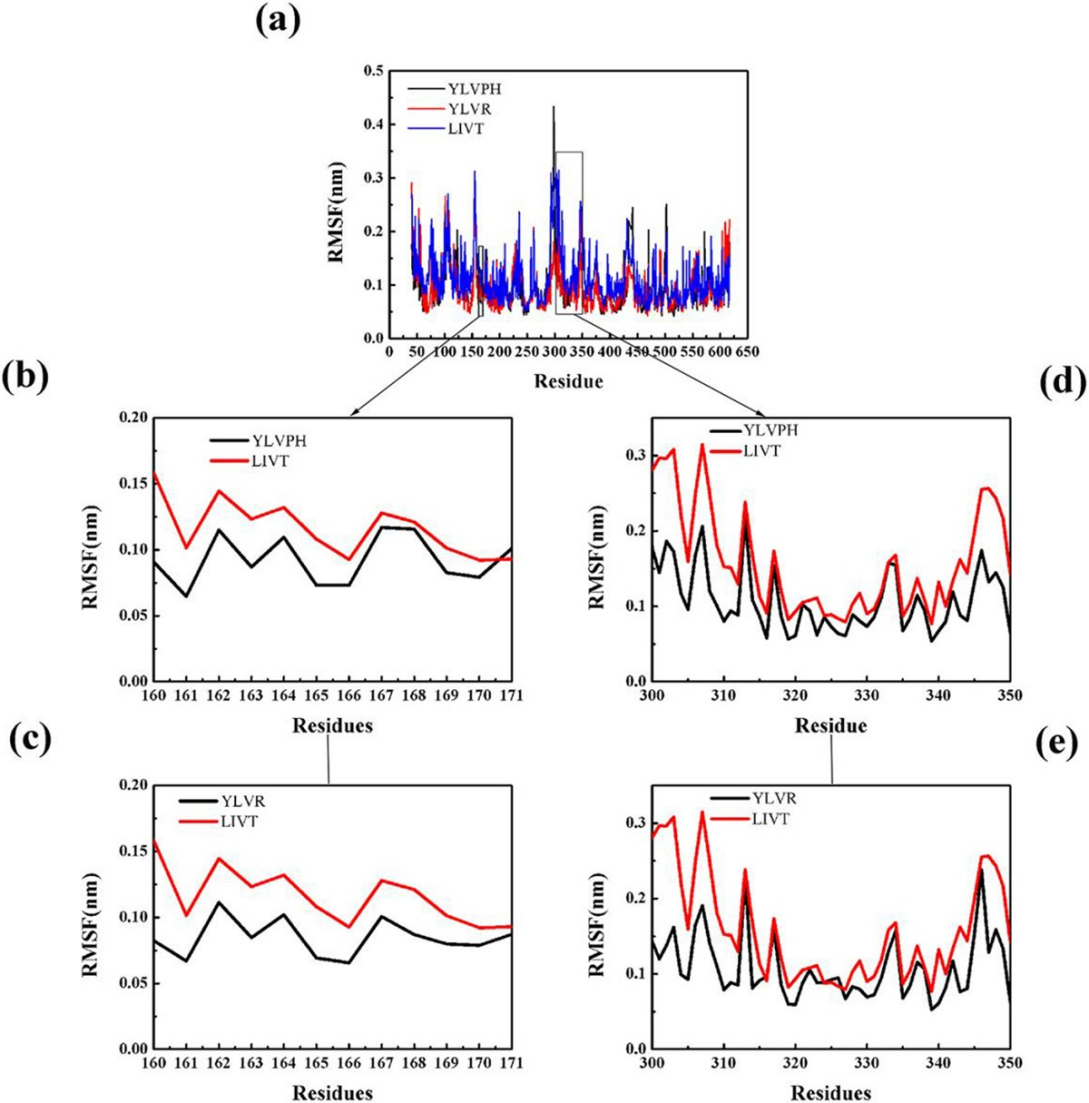
Result of RMSF of three inhibitor-protein complex systems during 400 ns MD simulation. (a) Total RMSF of three inhibitor-protein complex systems (YLVPH is shown in black, YLVR is shown in red, LIVT is shown in blue). (b-c) Comparison of the effects of different inhibitors on RMSF for α5. (d-e) Comparison of the effects of different inhibitors on RMSF for α10–12.

The structural flexibility of the three complexes was calculated by RMSF analysis (Fig 6). The Asp300-Val350 domain showed higher fluctuations in the ACE-LIVT complex compared to the other two complexes.

**Fig 7:**
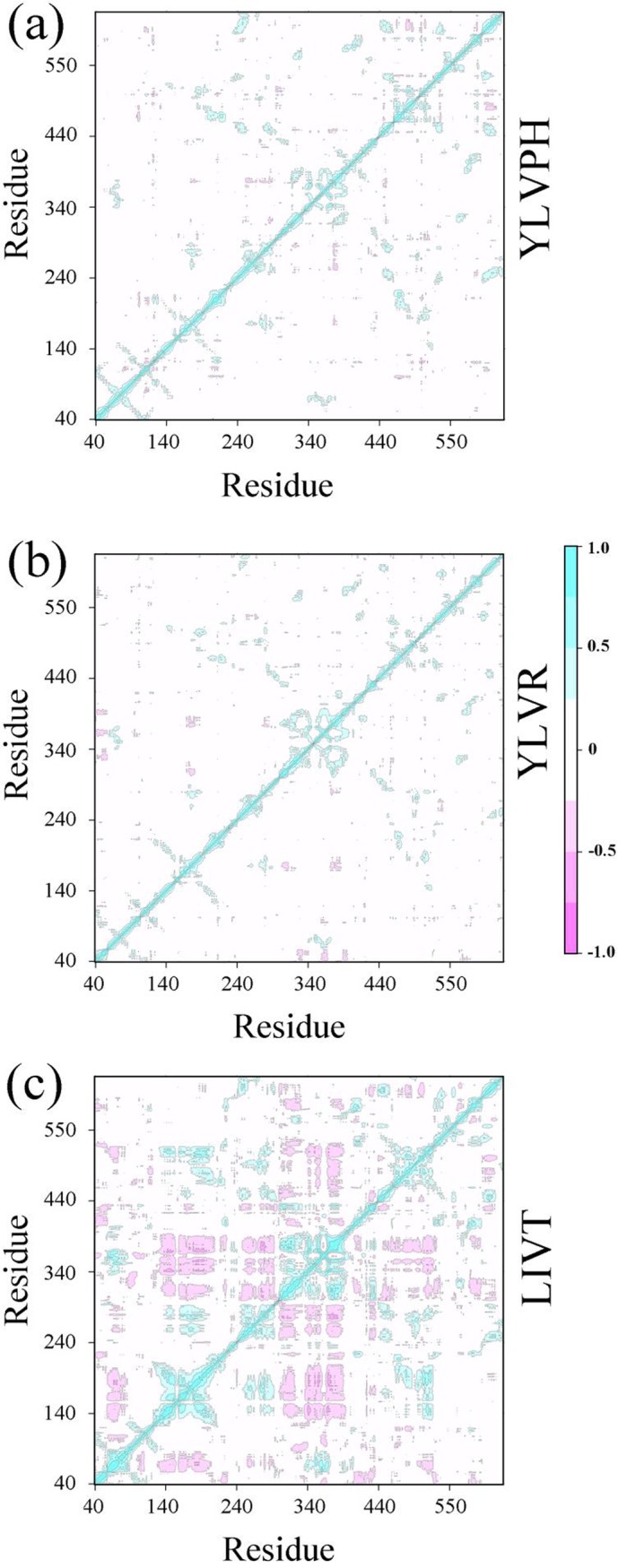
Results of covariance of three inhibitor-protein complex systems. The cross-correlation matrix maps for (a) ACE-YLVPH, (b) ACE-YLVR, and (c) ACE-LIVT systems. The positive regions, marked in cyan, indicate the strongly correlated motions of residues, whereas the negative regions, colored by pink, are associated with the anti-correlated movements.

**Fig 8:**
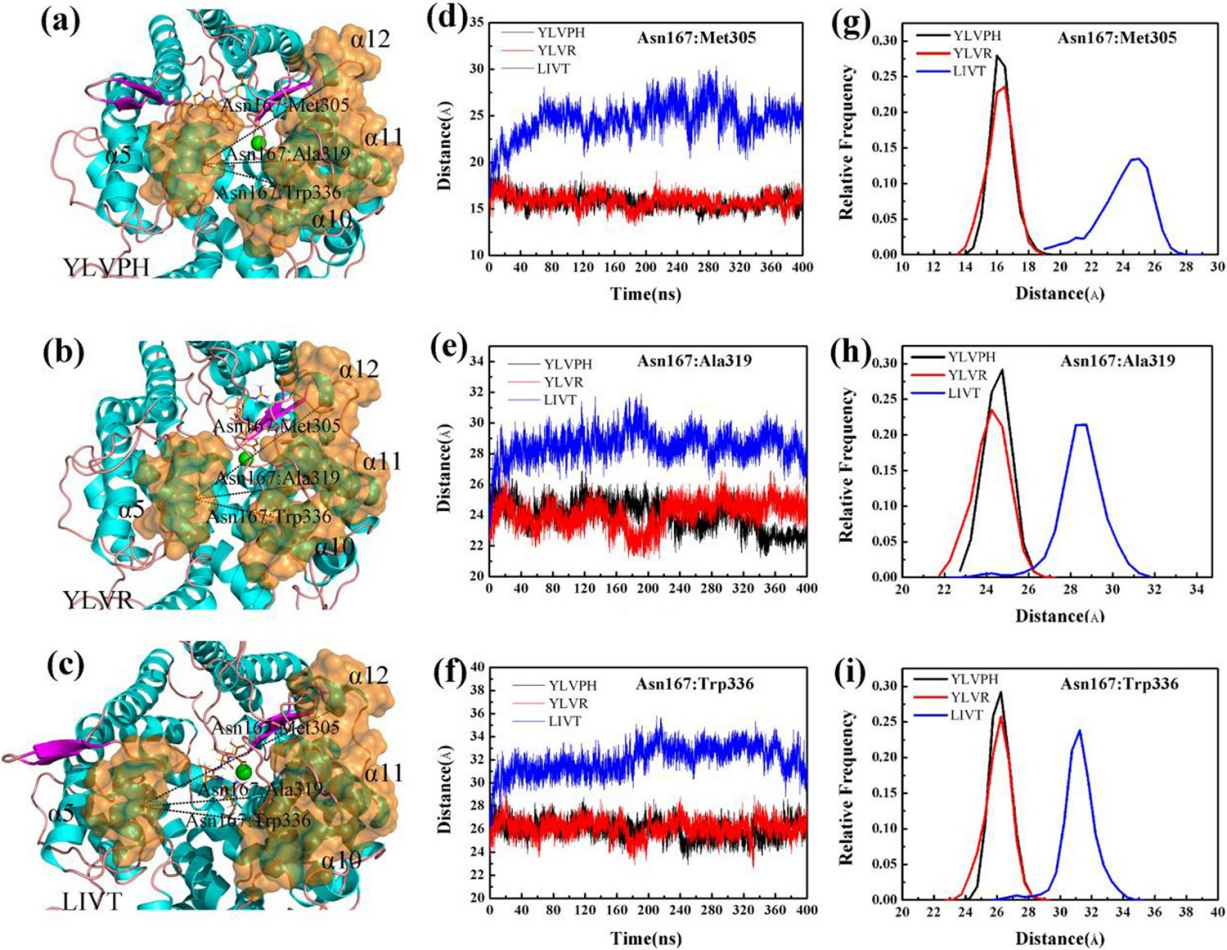
Distance variations betweenAsn167 and Met305, Asn167 and Ala319, and Asn167 and Trp336. (a-c) The relative positions between Asn167, Met305, Ala319 and Trp336 in ACE-YLVPH, ACE-YLVR, and ACE-LIVT complex systems. (d-e) Distance variationsbetweenAsn167 and Met305, Asn167 and Ala319, andAsn167 and Trp336 in three systems during 400 ns simulations. (g-i) Relative frequency between Asn167 and Met305, Asn167 and Ala319, and Asn167 and Trp336 in three systems.

The correlation matrix analysis can be used to strengthen the understanding of enzyme regions and can clarify the dynamic motion of ACE induced by binding by different inhibitors. The number of eigenvalues for the covariance matrix of the ACE was 1722. The cumulative variances of the first five eigenvectors are listed in Table 2. The first five eigenvectors of LIVT were found to represent at least 50% of the total motion, with the first two representing almost 35%. The covariance matrix maps of the three complexes are illustrated in Fig 7. The positive regions, represented by the color cyan, show the strongly correlated motions of the residues, whereas the negative regions, which are represented by the color pink, were associated with the anti-correlated movements. The diagonal was relatively highly correlated since it represented the variance of a residue with itself. In particular, LIVT binding to ACE can strongly induce a series of correlated and anti-correlated motions of ACE compared with the other inhibitors (Fig 7C). The matrix maps of the three systems indicated that significant motions, correlated or non-correlated, mainly occurred among the regions of residues 161–171 (α5) and 300–350 (containing α10, α11, and α12), respectively. Particularly, the motion in residues 300–350 attracted more attention near the active pocket of α13. The binding increased the correlated motion between residues 161–171 and 300–350, whereas the correlated motion of these domains became weak in the presence of YLVPH and YLVR. The results for the ACE-LIVT complex show that α5 may move away from α10, α11, and α12 (see Fig 8), thereby enlarging the active pocket, and may be useful for LIVT to slide in.

**Table 2:** Probability of PC1 to PC5 for YLVPH, YLVR, and LIVT.

System	Principle component (PC)	Probability
YLVPH		
	PC1	18.19%
	PC2	9.26%
	PC3	5.40%
	PC4	4.01%
	PC5	2.99%
YLVR		
	PC1	14.31%
	PC2	5.40%
	PC3	5.18%
	PC4	4.08%
	PC5	3.56%
LIVT		
	PC1	24.64%
	PC2	14.96%
	PC3	7.50%
	PC4	3.80%
	PC5	2.92%

**Fig 9:**
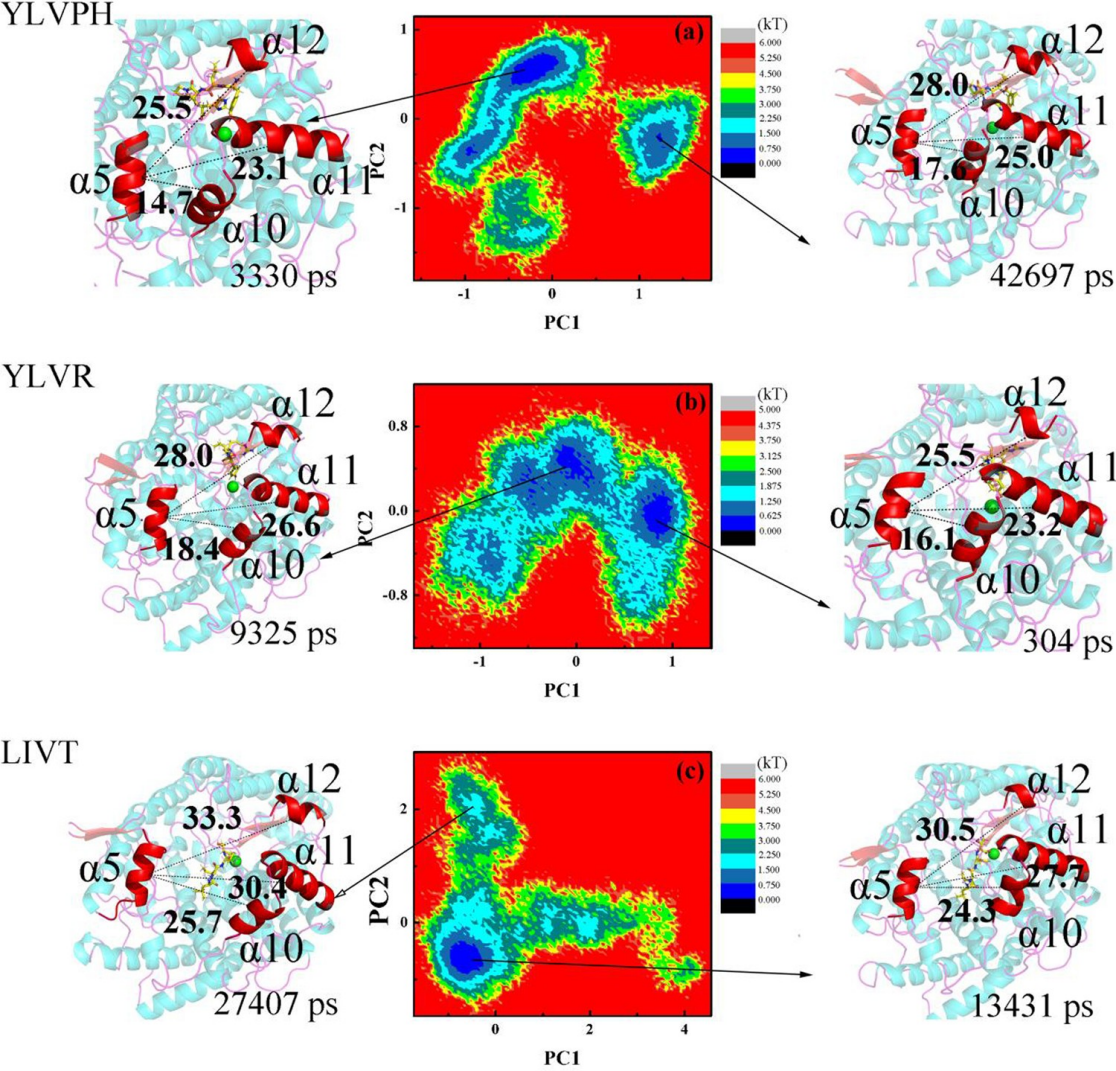
Result of principal component analysis (PCA) for three inhibitor-protein complex systems. Free energy landscape (FEL) and structures of the two most stable structures of the three systems. The lowest energy structures of PC1 (lift) and PC2 (right) are shown as a cartoon. The α5, α10, α11, and α12 helix are indicated in red. (a) ACE-YLVPH, (b) ACE-YLVR, and (c) ACE-LIVT.

Therefore, the projections for PC1 and PC2 are shown by the mode directions for each residue (Fig 9), which draw the dominant motions during the 400 ns MD and the lowest energy structures of PC1 and PC2 in the three complexes. From Fig 9, it can be observed that in the ACE-LIVT complex, residues 161–171 (α5) move away from residues 300–350 (containing α10, α11, and α12) either in PC1 or in PC2 compared with ACE-YLVPH and ACE-YLVR.

During three MD simulations, there were no stable hydrogen bonds interaction between inhibitors and ACE. From S2 and S3 Figs, the SASA of amino acids of ACE of three inhibitors were listed. Residues Val351 and His387 had higher scores in the ACE-YLVPH complex than that of other complex'. And so they may be the key residues for YLVPH binding.

In summary, LIVT binding to ACE may destroy the compactness of the protein, while YLVPH and YLVR can induce stronger and more centralized self-correlated motions in ACE. Moreover, these correlated and anti-correlated motions may be responsible for enlarging the active groove of ACE and were useful for inhibitor binding.

### Computational alanine scanning

**Fig 10:**
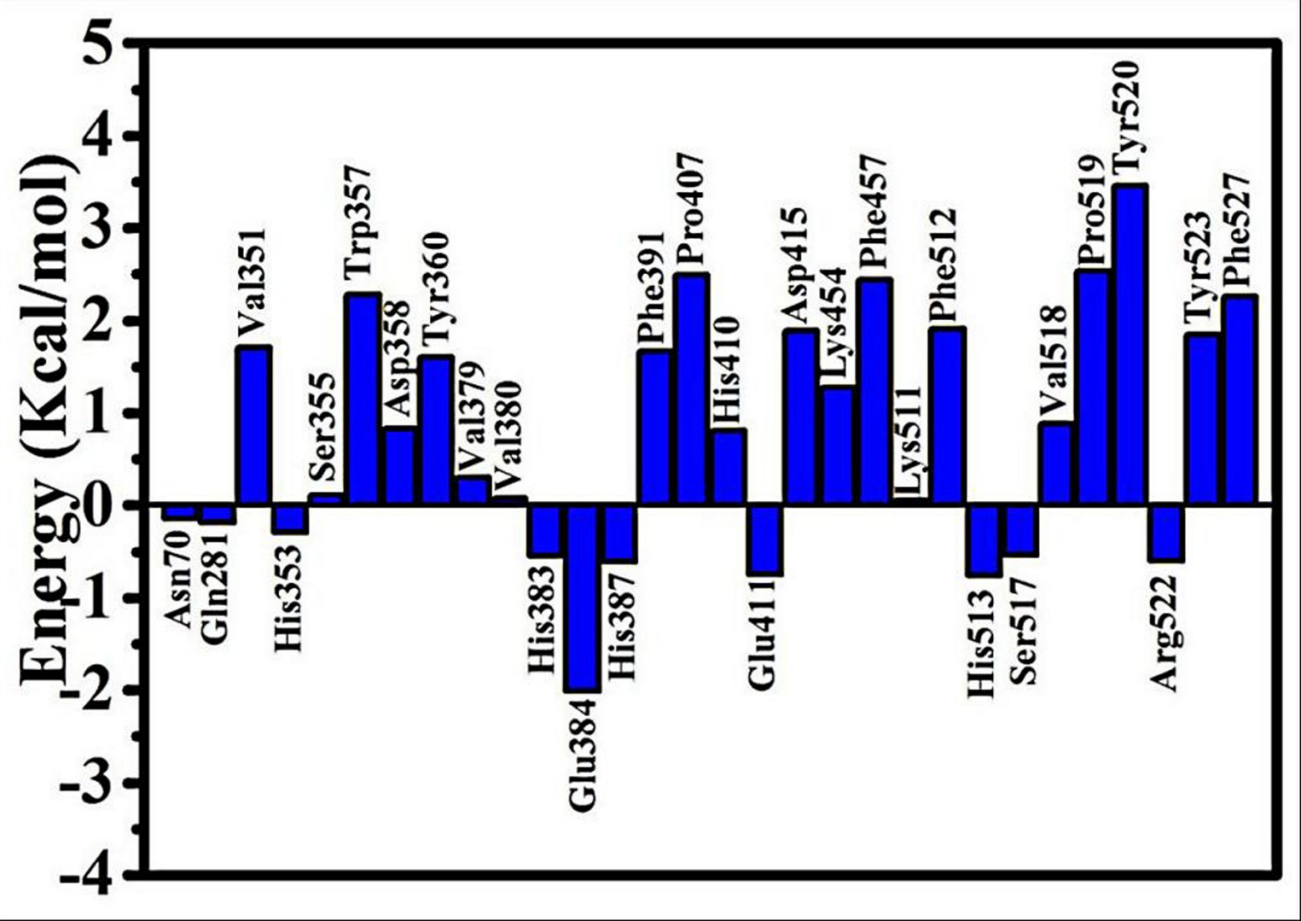
Computational alanine scanning of residue binding sites. The analysis was performed using the FoldX approach applied to conformational ensembles obtained from 400 ns MD simulations. Energetic binding hotspots correspond to residues for which alanine scanning resulted in a significant decrease in the binding free energy.

Computational alanine scanning using FoldX of the selected amino acid residues of the inhibitor binding site groove was carried out to elucidate the role of individual residues on the stability of the protein structure. In our research, the residues that surrounded the inhibitors (YLVPH, YLVR, and LIVT) were mutated to alanine to detect its influence on the stability of the protein structure. The results of the mutagenesis are presented in Fig 10. It can be observed that the Asn70, Gln281, His353, His383, Glu384, His387, Glu411, His513, Ser517, and Arg522 mutations could reduce the energy of ACE. Among them, the Glu384 mutations had the greatest influence on protein energy (-2.01 kcal/mol).

On the contrary, the Val351, Trp357, Tyr360, Phe291, Pro407, Asp415, Phe457, Phe512, Pro519, Tyr520, Tyr523, and Phe527 mutations could increase the energy of the protein, which may reduce the stability of ACE. In addition, the Ser355, Val379, Val380, and Lys511 mutations had almost no effect on the energy of ACE. The alanine scanning results suggested that the residues surrounding the inhibitor regulated its binding with inhibitors by influencing the energy of ACE.

## Conclusion

Theoretical study can simply explain the structural changes required for designing a new peptide that binds to ACE. In this study, we presented a systematic investigation of the structural basis and energetic profile of peptides that inhibit ACE by using a number of distinct but complementary molecular modeling methods, including MD simulations and computational alanine scanning, which was for the first time employed to explore the peptides to investigate the molecular mechanisms underlying ACE-peptide conformational changes and interactions. We conclude that the docking between ACE and the inhibitors conformed to the geometry of the tunnel, and these results can be used in future MD simulations. Furthermore, the results from the MD simulations of the three systems showed that the backbone movement of LIVT was more vigorous in Asp300-Val350 compared with that of YLVPH and YLVR, and the center-of-mass distance between Ala170 and Thr302 of LIVT changed significantly.

## Supporting information

S1 Table The docking results. (DOCX)

S1 Fig (a) The initial conformation of YLVPH. (b) The average conformation of YLVPH. (c) The final confirmation of YLVPH. (d) The initial conformation of YLVR. (e) The average conformation of YLVR. (f) The final confirmation of YLVR. (g) The initial conformation of LIVT. (h) The average conformation of LIVT. (i) The final confirmation of LIVT.
(TIF)

S2 Fig SASA scores for the three complexes. (TIF)

S3 Fig SASA scores for the three complexes. (TIF)
