# Supplementary material for: Structural and molecular basis of angiotensin-converting enzyme by computational modeling: Insights into the mechanisms of different inhibitors
Source: PLoS One. 2019 Apr 18;14(4):e0215609. doi: 10.1371/journal.pone.0215609 (PMC6472769; doi:10.1371/journal.pone.0215609)
Supplement: S1 Table — (DOCX) [file pone.0215609.s001.docx]

**Table s1 The docking results.**

| Complex (Protein-binder) | YLVPH | YLVR | LIVT |
| --- | --- | --- | --- |
| H-Bond | Ala352 | His353 | Ala356 |
|  | Glu411 | Ala356 | His353 |
|  | Tyr523 | His383 | His383 |
|  | His387 | His387 | His387 |
|  |  |  | His513 |
|  |  |  | Tyr523 |
| Vdw | His387 | Asn70 | Ser355 |
|  | His512 | Val518 | Glu411 |
|  | Ser355 | Trp357 | Tyr520 |
|  | Val518 | Arg522 | Gln281 |
|  | Trp357 | Val351 | Phe457 |
|  | Ser517 | Phe512 | Lys511 |
|  | Pro519 | Ser355 | Phe527 |
|  | Pro391 |  | Lys454 |
|  | His410 |  | Val379 |
|  | Arg522 |  | Val380 |
| ΔGbind(kcal/mol) | -7.45 k | -7.03 | -9.94 |
| Number of conformations in the first ranked cluster | 21 | 21 | 21 |
