# Supplementary figures and images for: Structural and molecular basis of angiotensin-converting enzyme by computational modeling: Insights into the mechanisms of different inhibitors
Source: PLoS One. 2019 Apr 18;14(4):e0215609. doi: 10.1371/journal.pone.0215609 (PMC6472769; doi:10.1371/journal.pone.0215609)

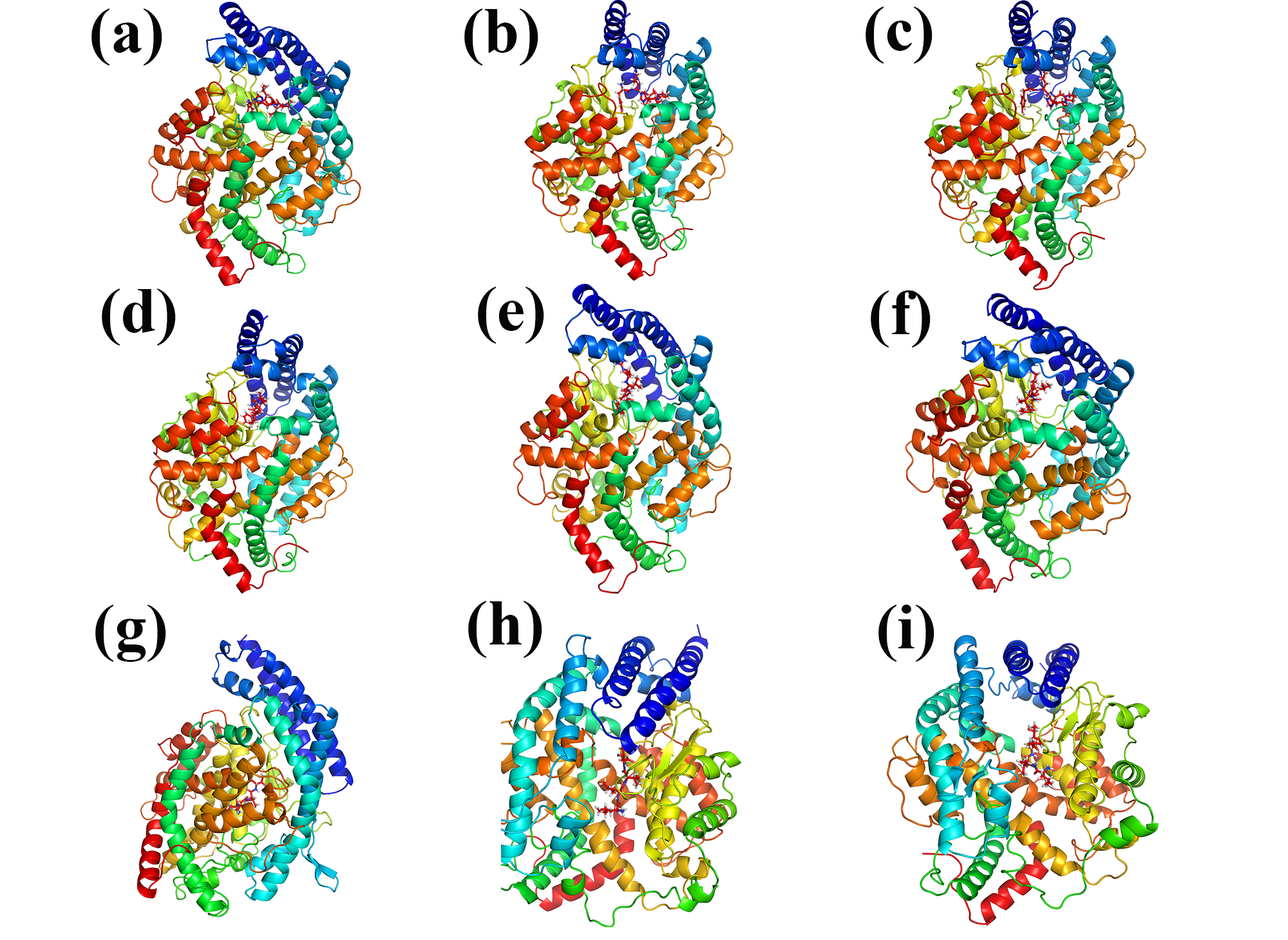

Supplement: S1 Fig — (a) The initial conformation of YLVPH. (b) The average conformation of YLVPH. (c) The final confirmation of YLVPH. (d) The initial conformation of YLVR. (e) The average conformation of YLVR. (f) The final confirmation of YLVR. (g) The initial conformation of LIVT. (h) The average conformation of LIVT. (i) The final confirmation of LIVT. (TIF) [file pone.0215609.s002.tif]

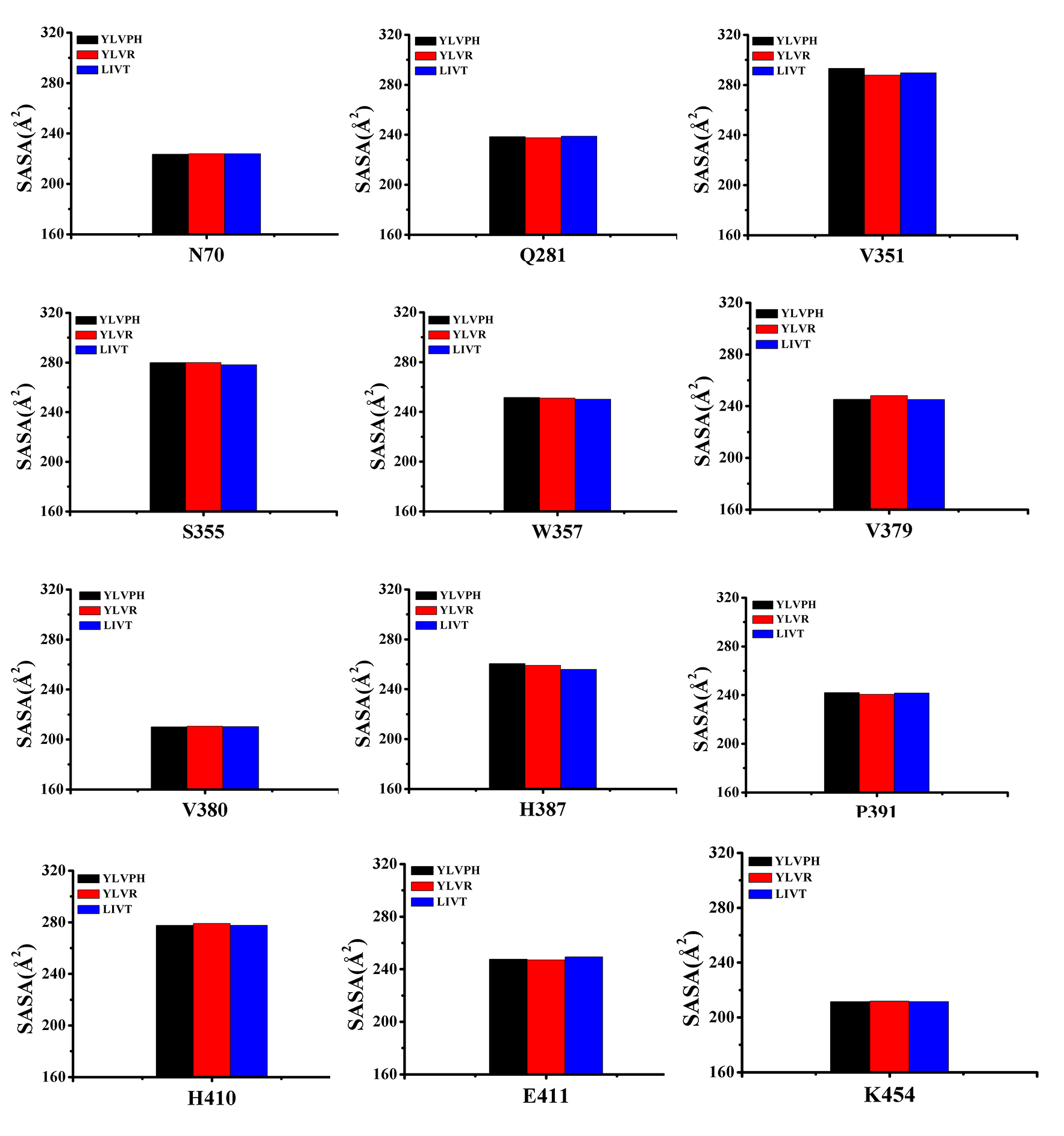

Supplement: S2 Fig — (TIF) [file pone.0215609.s003.tif]

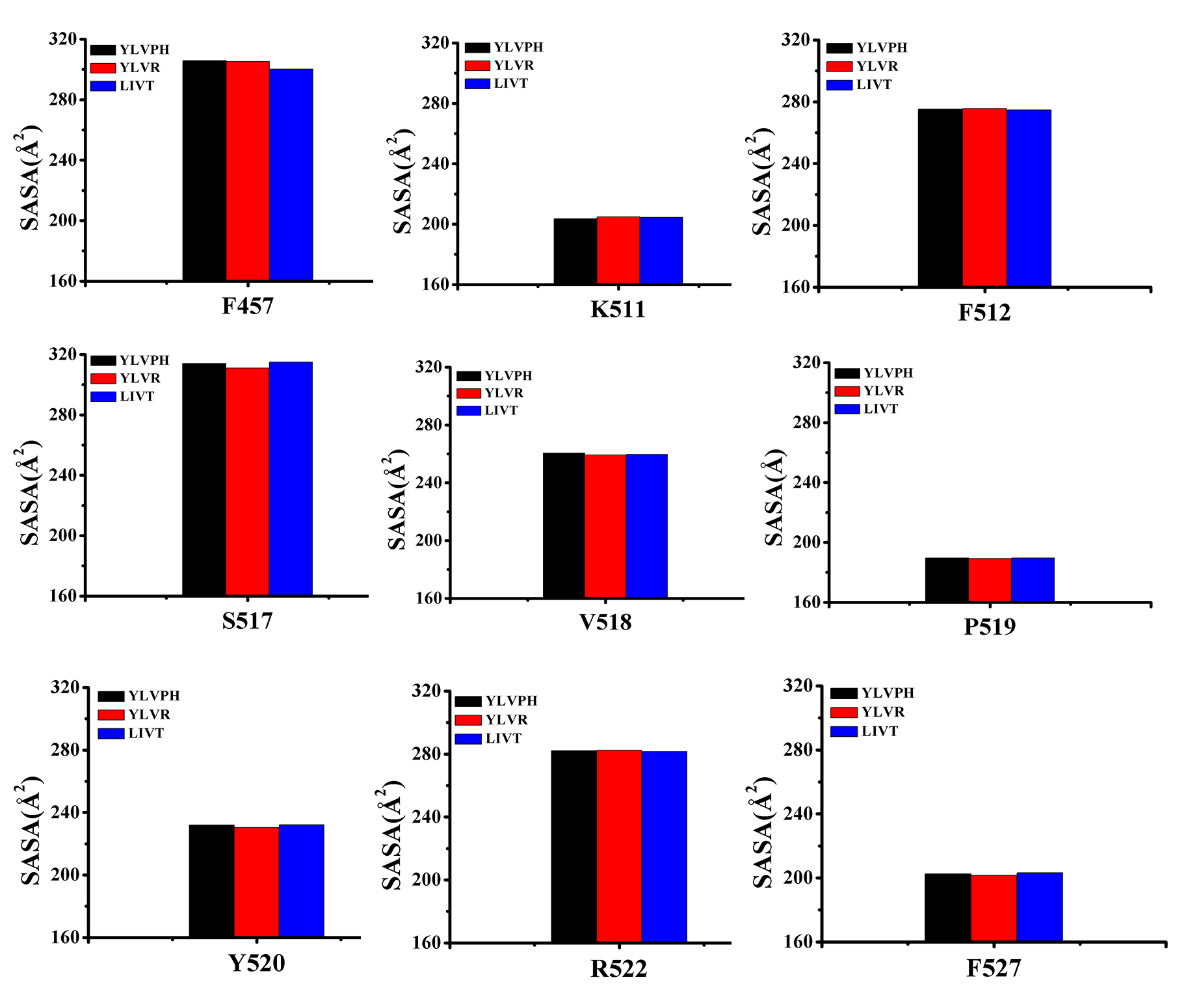

Supplement: S3 Fig — (TIF) [file pone.0215609.s004.tif]
